# First confirmed identification of a male Asian longhorned tick (Ixodida: Ixodidae) in the United States

**DOI:** 10.1093/jme/tjaf040

**Published:** 2025-03-27

**Authors:** Sara F Margrey, James W Mertins, Leeanne C Garrett, Richard Gary, Risa Pesapane

**Affiliations:** Ohio Department of Health, Columbus, OH, USA; USDA APHIS, Veterinary Services, Diagnostics and Biologics, National Veterinary Services Laboratories, Ames, IA, USA; Ohio Department of Health, Columbus, OH, USA; Ohio Department of Health, Columbus, OH, USA; Department of Veterinary Preventive Medicine, College of Veterinary Medicine, The Ohio State University, Columbus, OH, USA; School of Environment and Natural Resources, College of Food, Agricultural, and Environmental Sciences, The Ohio State University, Columbus, OH, USA

**Keywords:** invasive species, veterinary health, Ohio, gynandromorph

## Abstract

*Haemaphysalis longicornis* Neumann, the Asian longhorned tick (ALT), has quickly established widespread invasive populations in the United States since its first at-large detection in 2017. Until recently, no male ALT has been verifiably collected in the United States, despite examinations of tens of thousands of individual specimens, thus affirming that the US incursion was founded by ticks from a parthenogenetic source population of ALT. This report documents the first validated male ALT specimen in the United States, collected in May 2023 on a cattle farm in Gallia County, Ohio. This specimen shows morphological signs of gynandromorphism in its palps, hypostome, genitalia, and anal aperture.

## Introduction

**Fig. 1.**
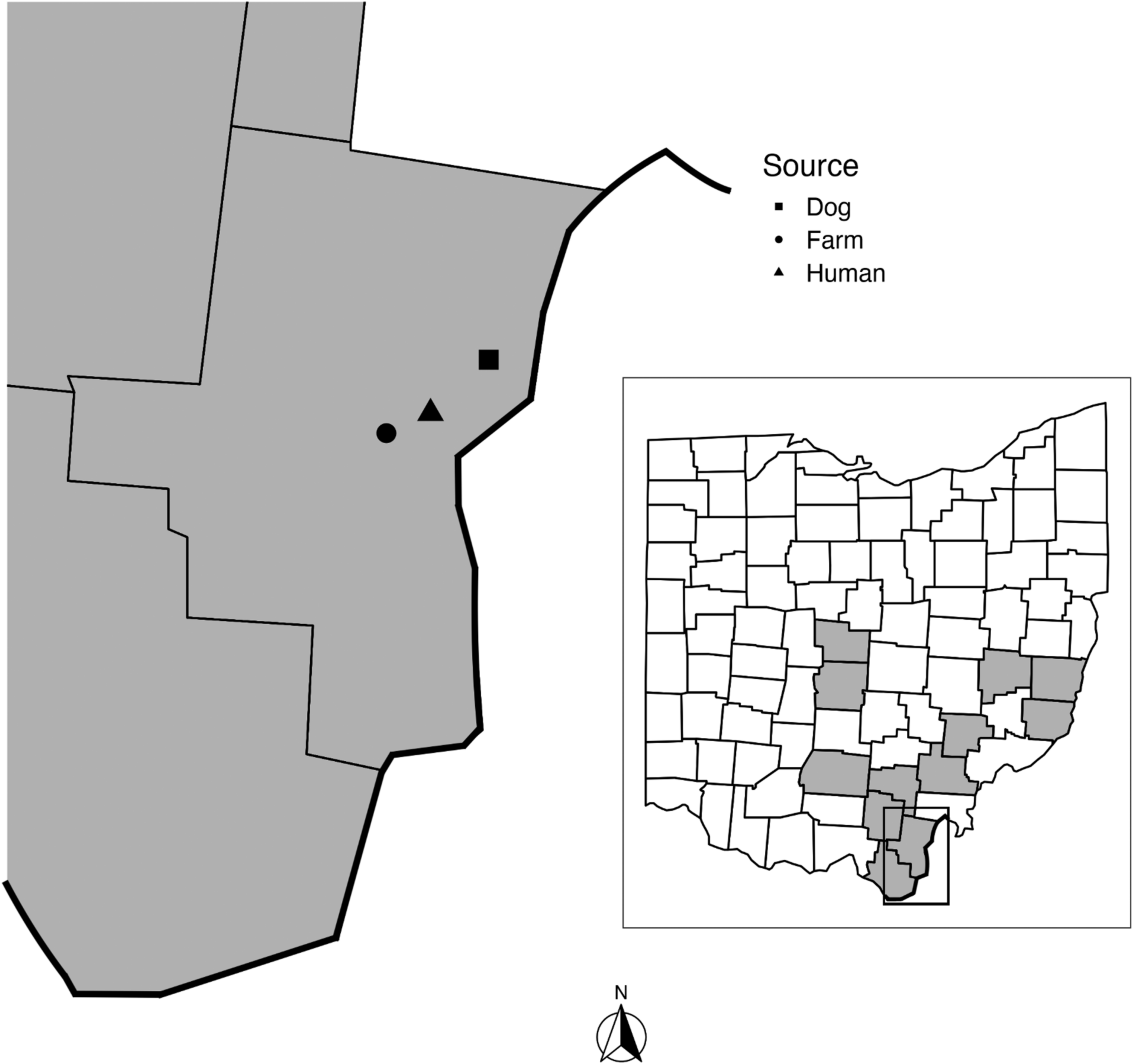
Approximate sites of *Haemaphysalis longicornis* collections in Gallia County, and (inset) 12 Ohio counties with *H. longicornis* reports (2010 to 2023, gray). Bold line indicates Ohio border with West Virginia, others are Ohio county lines. The person bitten by a *H. longicornis* had movement within a 32-km radius of the Gallia County Health Department office in the 3 d before submitting the tick; the health department building serves as an approximate indicator location.

The Asian longhorned tick (ALT), *Haemaphysalis longicornis* Neumann, is native to East Asia but has established invasive populations in the Australasian and Western Pacific Regions, and more recently, in the United States (Hoogstraal et al. 1968, Beard et al. 2018). Although the ALT was first detected at large in the United States in 2017 on a New Jersey sheep, retrospective evaluation of archived museum specimens revealed that the tick was actively but covertly present in the United States as early as 2010 in West Virginia (Beard et al. 2018, Rainey et al. 2018). Subsequent to 2017, active surveys for the ALT quickly detected its expanding invasive range in the United States, and as of August 2024, it had been reported in 21 states (USDA 2024). The first verified collection of an ALT in Ohio was from a stray dog in the city of Gallipolis in Gallia County in May 2020 and subsequent detection on a person in June 2022 (Fig. 1) (USDA 2024).

Invasive ALT populations in the United States are exclusively parthenogenetic (Egizi et al. 2020), although endemic populations in Asia are variously bisexual or parthenogenetic (Heath 2016). In parthenogenetic populations, because every ALT individual is a reproductive female, this tick has an unusual capacity to invade new environments and establish rapidly growing populations. In Old World ALT parthenogenetic populations, males are known to occur rarely (Bremner 1959, Hoogstraal et al. 1968), and to date in the United States, only 1 resident male tick is reported (Rainy et al. 2018), although no voucher specimen exists to support the claim.

This report documents the first verifiable ALT male specimen collected in the United States, along with the circumstances of its collection and its anomalous morphological features.

## Materials and Methods

In mid-April 2023, the owner of an approximately 60-head cattle herd grazing on un-mowed pasture in Gallia County, Ohio, found a 6-week-old Charolais heifer calf unable to rise and showing clinical signs of inappetence and weakness. The herd had no travel history nor introduction of new cattle. The herd owner found 3 ticks on the calf and routed them through the Gallia County Health District to the Ohio Department of Health (ODH) for identification. A public health entomologist examined the ticks under a 1.8 to 11× zoom stereomicroscope and identified them as 3 female ALTs (2 partially engorged and alive, 1 fully engorged and dead) using pictorial keys (Keirans and Litwak 1989, Egizi et al. 2019).

On 10 May 2023, ODH officials (entomologist and state public health veterinarian) visited the farm to survey ticks on cattle and in the environment. The cattle herd was evaluated for ticks and treated with pour-on permethrin (ProZap Backrubber and Pour-On Xtra, Neogen, Lansing, Michigan). Ticks observed during physical examination were heavily infesting the heads, ears, and perianal regions of all cattle. Ticks were collected from each animal with forceps or tick removal tools (Ticked Off Tick Remover, Ticked Off Inc., Dover, New Hampshire). The ODH collected ticks from the affected pasture by dragging 1-m^2^ white flannel cloths on the ground, with inspection stops every 10 to 15 m. All ticks were removed with forceps and stored in vials with 70% ethanol. This sampling covered approximately 1,500 m^2^ of the roughly 25.8-ha pasture, focused on edge habitat. An ODH entomologist examined and identified ticks collected from both the cattle and pasture. All unfed ticks were saved for identification confirmation and pathogen testing.

The US Department of Agriculture’s National Veterinary Services Laboratories (NVSL) confirmed species identification of a putative male specimen by reference to the descriptions, illustrations, and keys in Hoogstraal et al. (1968), Yamaguti et al. (1971), and Egizi et al. (2019). Initial examination and identification were made under a dissecting microscope, but later, the specimen was studied and photographed with a Hitachi TM3030 scanning electron microscope (Hitachi America, Ltd, Santa Clara, California).

## Results

Initial ODH laboratory examinations of field-collected samples identified a total of 521 ticks. Both environmental and cattle samples comprised the same 3 tick species, *Dermacentor variabilis*, *Amblyomma americanum*, and *H. longicornis*. Among the ticks from both sources combined, ALTs composed the majority (437 of 521 individuals), including all 3 active life stages (319 adults, 117 nymphs, and 1 larva). The majority of identified ALTs (358, 81.9%) came from cattle, including 318 adults and 40 nymphs. Initial laboratory examination tentatively identified 1 adult male ALT specimen in the cattle collections. Subsequently, The Ohio State University preliminarily confirmed the specimen as male based on morphological characteristics and forwarded the tick to the NVSL for final confirmation, detailed study, and archiving.

**Fig. 2.**
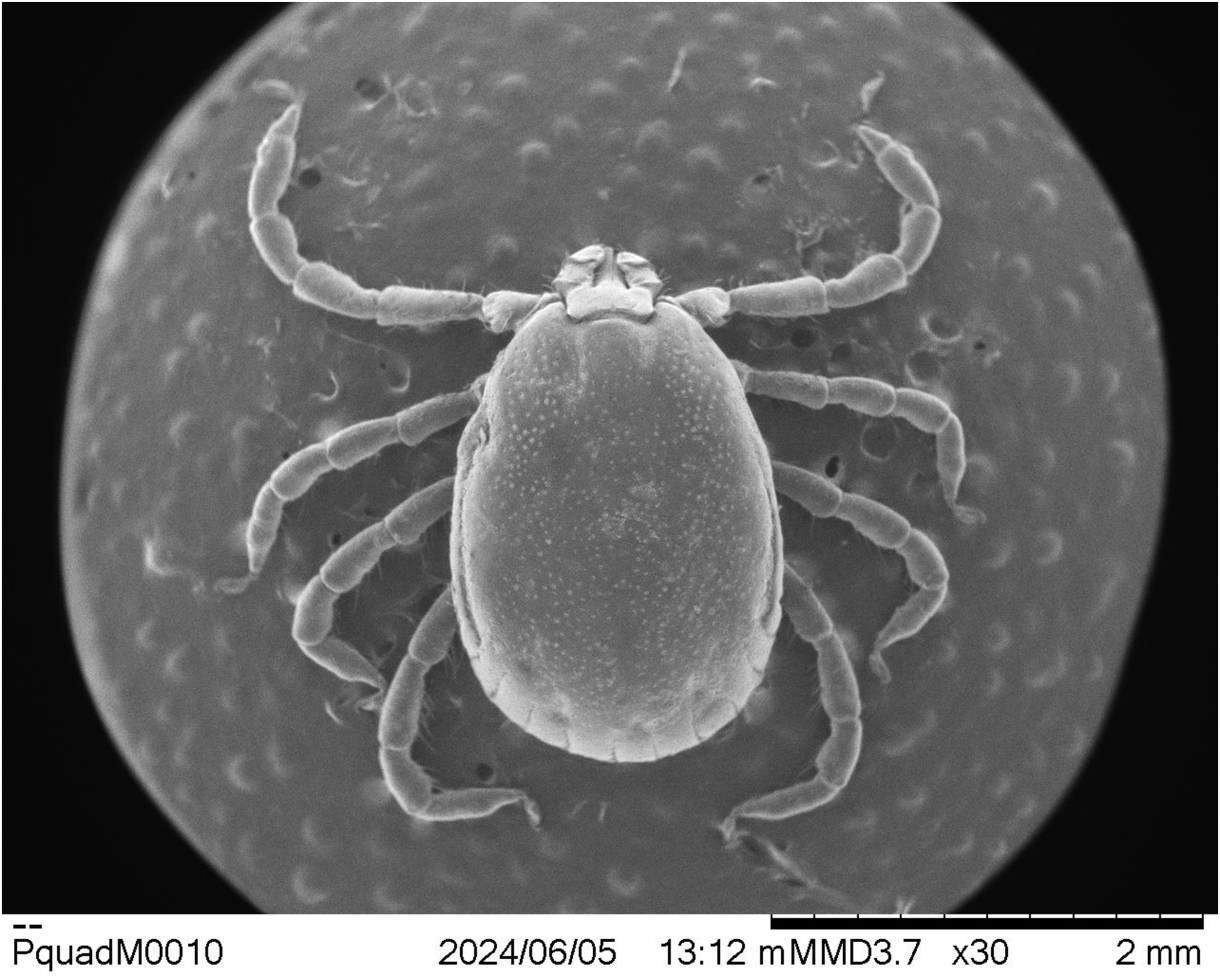
Male *Haemaphysalis longicornis* (dorsal habitus) from a cow in Gallia County, Ohio (May 2023).

**Fig. 3.**
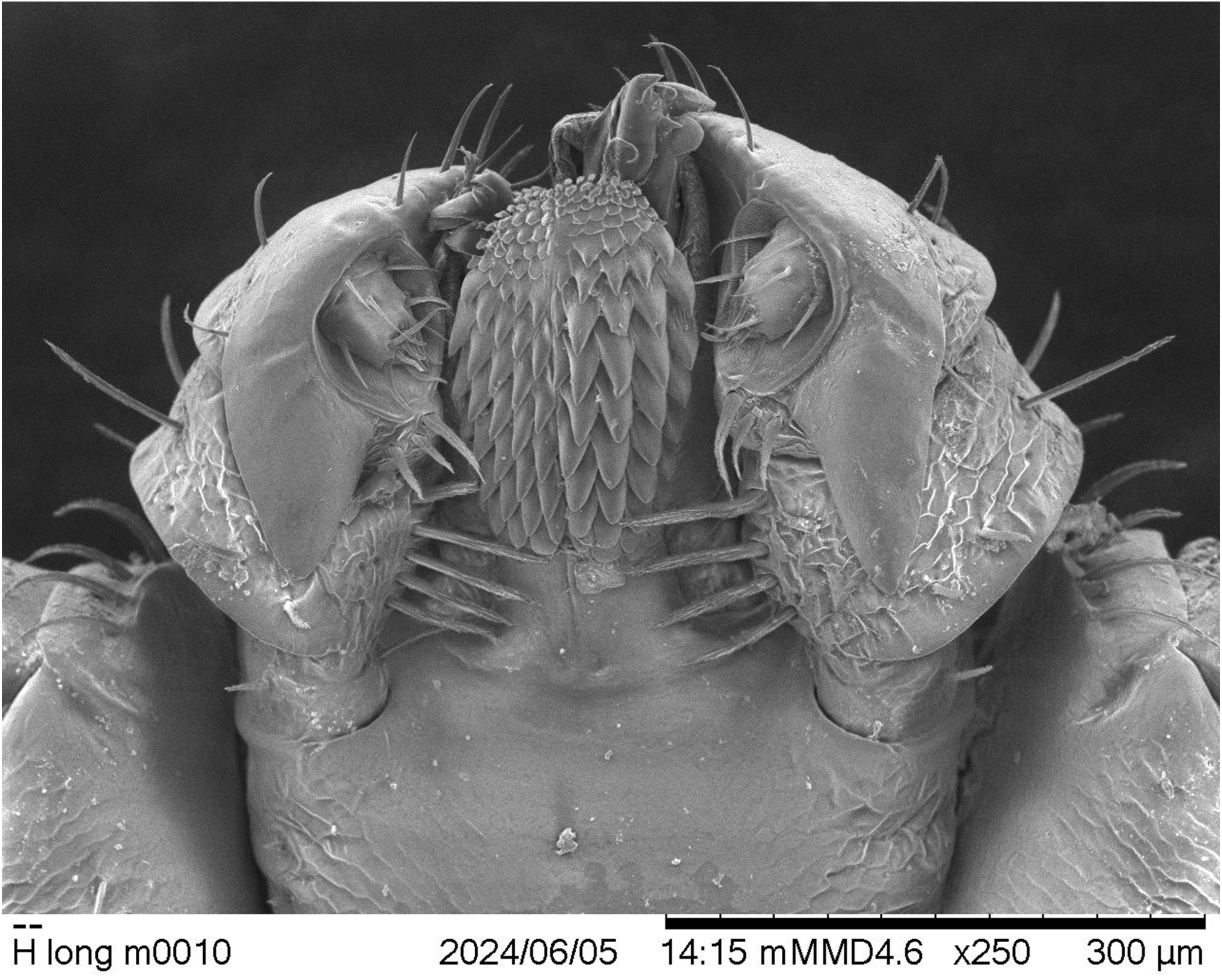
Dysmorphic capitulum (ventral aspect) of the Ohio male *Haemaphysalis longicornis* specimen. Note asymmetrical hypostomal dentition, and structural asymmetries of the palps.

During the process of confirming species identification, the NVSL entomologist noted a number of morphological anomalies, asymmetries, or teratologies in the subject male ALT specimen. Although most diagnostically important anatomic features of the specimen were nearly normal and usable for the purpose of species identification (Fig. 2), some structures showed minor anomalies. For example, the overall size and shape of 1 palp are different from those of the other, including slight symmetrical differences in both the dorsal and ventral spurs of palpal article III. This disparity is particularly notable ventrally, where the spur on the tick’s left-side palp is longer and narrower than the one on the right side (Fig. 3). Moreover, the often-diagnostic internal ventral setal rows on palpal articles II are asymmetrical as well. The tick’s right-side palp bears 5 such setae (the tip of the most distal seta is broken off), but the left-side palp has only 4 longer and stouter setae. Hoogstraal et al. (1968) described and showed 4 of such setae on both palps, based upon observation of a single Australian male specimen, but in their observed Australian female specimens, the described number was 4 or 5. Four setae is the number used in other descriptions of Australian males (Barker and Walker 2014), as well, although a line drawing of a Japanese male capitulum in Yamaguti et al. (1971) shows 4 such setae on 1 palp and 5 on the other; Egizi et al. (2019) show 5 setae on each Korean male palp in an SEM image, but 6 on each Korean female palp; and Dönitz (1905) describes the typical number as 5 in both sexes of his Japanese specimens.

Most notably, the ventral aspects of the 2 halves of the hypostome on this specimen are clearly offset from each other, and their dentitions are not mirror images (Fig. 3). Initially, under a dissecting microscope, this disparity was substantially obscured by the largely enveloping palps, but during the SEM process, differences were easily observable. The dental formula of this specimen is 4.5/5, with the individual denticles on the tick’s left side clearly larger than those on the right. Most of the hypostomal rows on the left side show 4 denticles each, but 3 rows in the distal half of the hypostome each have a smaller fifth denticle medially. Male ALTs are supposed to have a formula of 5/5, and females the same, or rarely, 5/4 or 5/6 (Dönitz 1905, Hoogstraal et al. 1968, Yamaguti et al. 1971, Egizi et al. 2019). The left side of the hypostome is slightly longer than the right side, and the reduced-size denticles composing the hypostomal corona are also different from each other on the 2 halves.

**Fig. 4.**
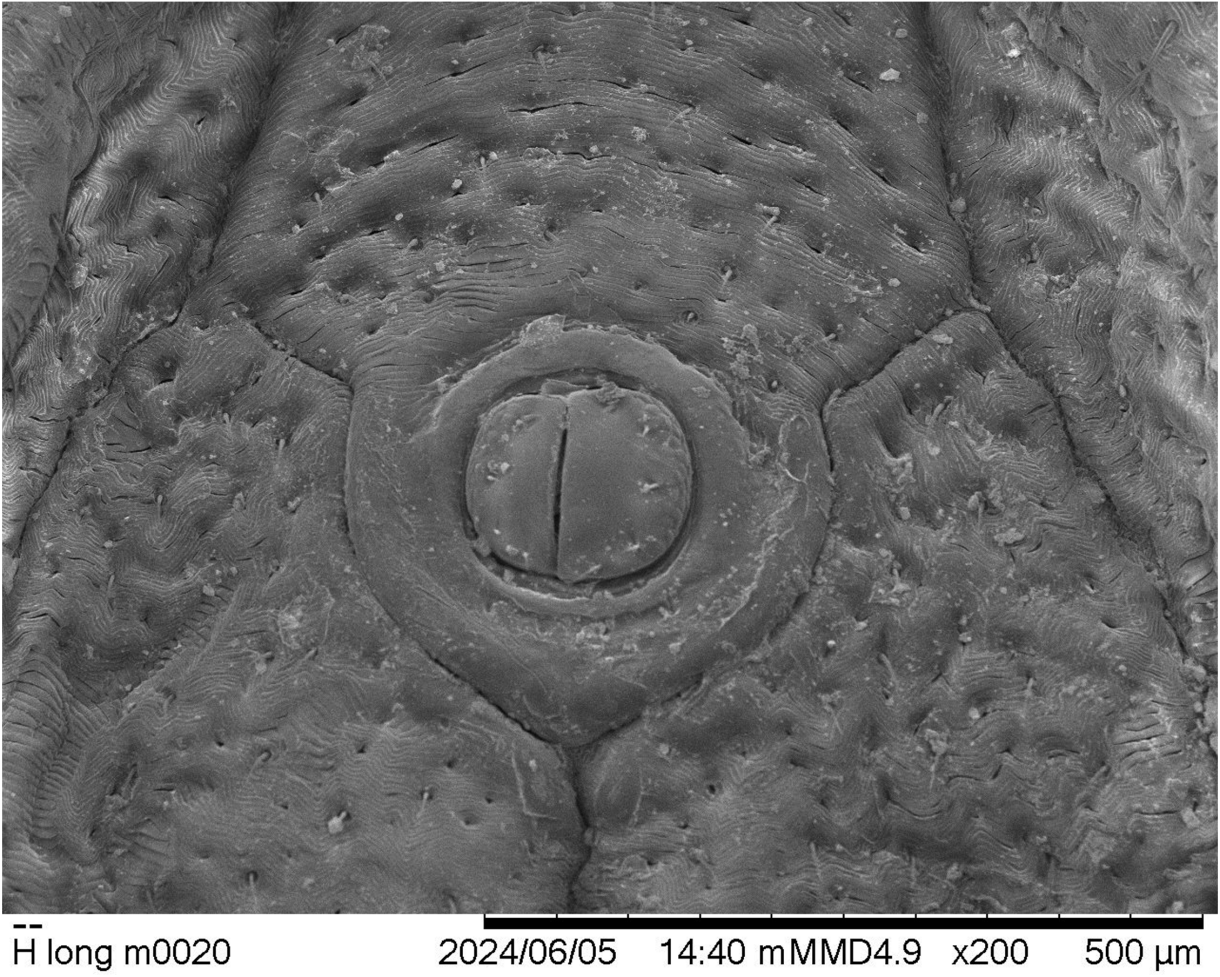
*Haemaphysalis longicornis* male from Ohio; anal field showing asymmetrical anal valves.

**Fig. 5.**
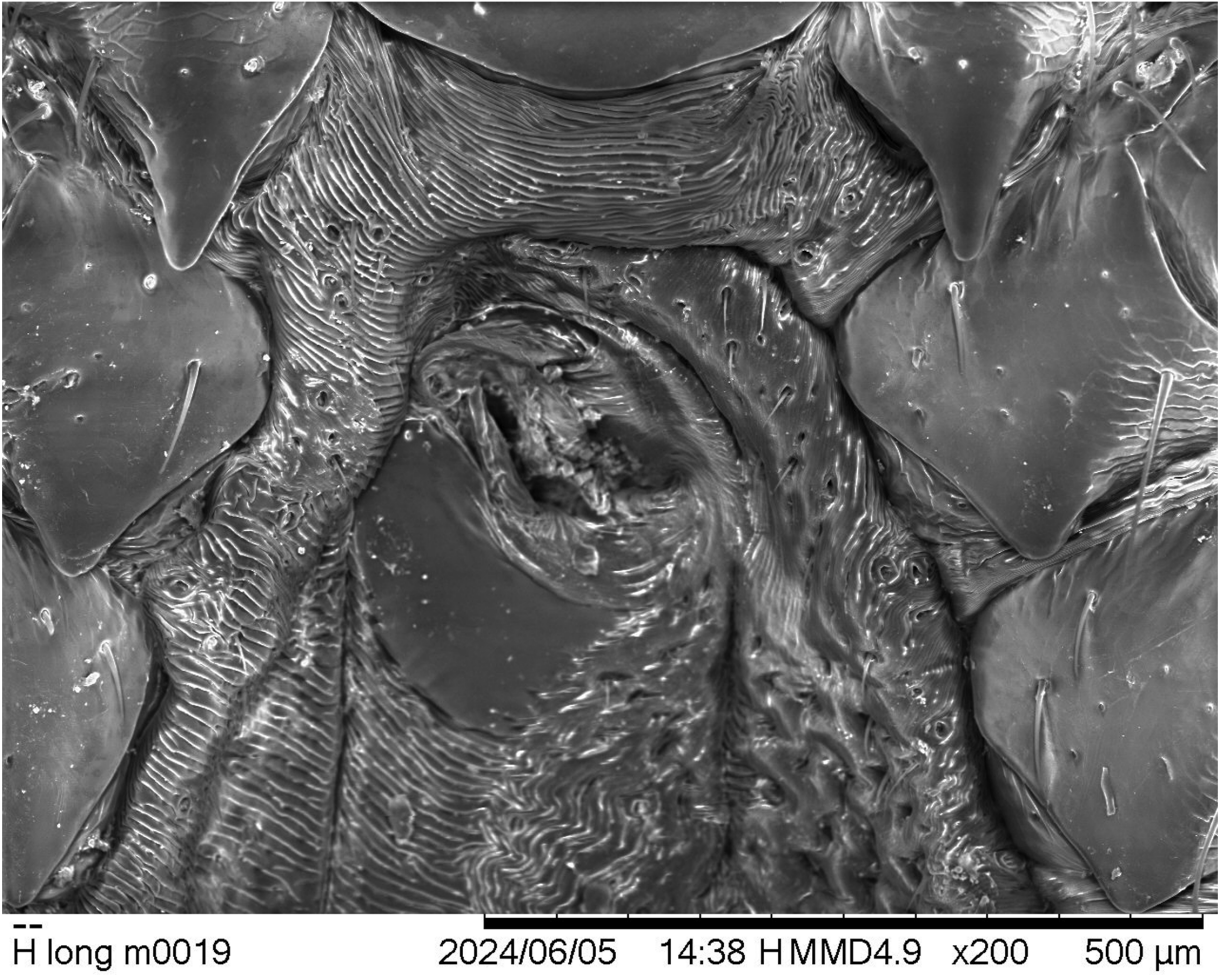
*Haemaphysalis longicornis* male from Ohio; genital area showing distorted orientation and dysmorphia.

Two other morphological features of this male tick specimen are notable. The external valves of the anal aperture are asymmetrical, with the valve on the tick’s left side about one-fourth larger than its counterpart (Fig. 4). And finally, the genital aperture is abnormally developed, based upon the illustrations of Hoogstraal et al. (1968). It seems to show the larger dimensions typical of male *H. longicornis* genitalia (compared to those of the female genital structures), but its orientation is twisted from what would be normal, and its component parts are muddled and only partly distinguishable (Fig. 5).

## Discussion

This report documents the first collection, identification, and confirmation of a male ALT in the United States. Although Rainey et al. (2018) reported the collection of a male ALT in New Jersey, this putative specimen was later not retrievable and was unavailable for reexamination and confirmation, leaving its initial, first-in-the-US report unverified.

Published data on the prevalence of males within Old World populations of *H. longicornis* are few (Tsunoda 2014, Chong et al. 2022). Kitaoka (1961) and Hoogstraal et al. (1968) stated that sex ratios in Asian bisexual populations vary greatly, citing male:female ratios of 11:21 and 83:44 as examples, respectively. Parthenogenetic populations of ALTs rarely produce males, with a small number of males (1 in 400) occasionally found in larger populations of females in Australia (Bremner 1959, Hoogstraal et al. 1968). Additionally, Oliver et al. (1973) observed a male:female ratio of 1:1,500 in progeny of an ALT lab colony. Observations in Ohio found only 1 male out of 319 ALT adults collected on the infested farm, although this small sample may not be broadly representative of the true prevalence of males in US ALT populations. This discovered tick is the only known and confirmed male specimen out of many thousands of ALTs collected and observed in the United States, a fact that still supports the parthenogenetic status of the US population.

Presence of a single male specimen in the parthenogenetic US population is unlikely to influence ALT population genetics or reproductive dynamics in the United States. Wherever they occur, parthenogenetic ALT populations are consistently successful and persistent either with or without the occasional occurrence of male specimens (Hoogstraal et al. 1968). Furthermore, Bremner (1959) demonstrated the absence of spermatozoa in males within parthenogenic Australian populations. This observation suggests that any males arising in the parthenogenetic US ALT population may be similarly sterile and therefore contribute nothing to local genetic diversity or reproductive success.

We report the first verifiable male in the United States in Gallia County, Ohio, where there have been multiple reports of ALT in relatively close proximity since 2020 (Fig. 1). The presence of a male in this established population may reflect a larger population size and potentially a longer history of ALT in this area, which could have increased the probability of a male occurring. Indeed, ALT were present in the neighboring state of West Virginia as early as 2010 (Beard et al. 2018). However, it is important to note that many areas with long histories of ALT reports have not produced males, highlighting the uncertainty surrounding this male’s emergence. Given the reported low frequency of males in parthenogenetic ALT lineages and excessively large population sizes, it is also possible males were simply missed at these other locations.

Assessment of observed morphological anomalies in the male Ohio tick specimen strongly suggests that it probably is, in fact, mildly gynandromorphic (metagynandromorphism, according to Campana-Rouget 1959). Context for this hypothesis is best provided by comparison of the specimen to illustrations of typical ALT male and female morphologies in Hoogstraal et al. (1968). In dorsal aspect (Fig. 2), little is visible to suggest that the Ohio specimen is not a male, but ventrally (Fig. 3), several visible bilateral asymmetries seem best explained by assigning them to Hoogstraal’s previously illustrated sexual dimorphisms. Essentially, the tick’s right-side palp, right-half hypostome, and right anal valve compare most closely to those of a male ALT, whereas their left-side counterparts compare more favorably to those of a female tick. The specimen’s genital area seems basically male-like, but its twisted orientation and abnormally developed components suggest some sort of developmental anomaly and almost certain reproductive dysfunctionality. Gynandromorphism in *H. longicornis* was reported previously only once, in laboratory-reared parthenogenetic ticks originating from northern Japan (Oliver et al. 1973).

In light of the demonstrated rare occurrence of male ALTs in US populations, and the singularity of this first example from Ohio, the specimen has been permanently retained as a voucher (NVSL accession no. 23-021953, case no. T23-750) without compromise in the NVSL parasitology reference collection. Genomic analysis of female ticks in its collected cohort is planned and will be reported separately.

## Notes

Disclaimer: Mention or listing of brand names or specific commercial products is solely for informational purposes and does not imply endorsement by the agencies employing the various coauthors, nor discrimination against similar brands or products not mentioned.
